# 

**Published:** 1877-12

**Authors:** 


### Books and Pamphlets Received.

Lectures on Fever. By Alfred L. Loomis, A. M., M.D. New York: Wm. Wood & Co., 1877. Buffalo: H. H. Otis.

Transactions of the New York Pathological Society. Vol. II. New York: Wm. Wood & Co., 1877. Buffalo: H. H. Otis.

The Culture of Beauty. By J. S. Sozinskey, M. D., Ph. D. Philadelphia: Allen, Lane & Scott, 1877.

Cutaneous and Venereal Memoranda. By H. G. Piffard, A. M., M. D. and G. H. Fox, A. M., M. D. New York: Wm. Wood & Co., 1877. Buffalo: H. H. Otis.

The Popular Science Monthly, December, and Supplement No. VII.

Diseases of the Nasal Cavity. Dr. Carl Michel. Trans. by E. L. Shurly, M. D., and C. C. Yemans, M. D.

The Sanitary Condition of Portland. By Frederick H. Gerrish, M. D. Portland: Stephen Berry, 1877.

What Anæsthetic shall we use? By Julian J. Chisolm, M. D.

The Safety of Ship and Those who Travel in them. By John M. Woodworth, M. D. Cambridge: Riverside Press, 1876.

Report on Dermatology. By Lunsford P. Yandell, Jr., M. D. Louisville: J. P. Morton & Co., 1877.

The Columbia Hospital. Reprint from Richmond & Louisville Med. Journal. November No.

Lecture on Medical Teaching in the University of Michigan. By A. B. Palmer, A. M., M. D.

Transactions of the Kansas Medical Society. Lawrence Journal Printing Establishment.

Seventh Annual Report of the Free Medical and Surgical Dispensary of Buffalo. October, 1877.

Circular of the Albany Eye and Ear Association, 1877.

Report of Eye and Ear Infirmary of Buffalo, 1877.

The following Reprints from the Transactions of the Am. Medical Congress:

- Views of Venereal Sores. By F. J. Bumstead, M. D.
- Medical Jnrisprudence. By S. E. Chaillé, A. M., M. D,
- Treatment of Aural Diseases. By Albert H. Buck, M. D.
- Medical Biography. By J. M. Toner, M. D.
- Quarantine with Reference to Cholera and Yellow Fever. By John M. Woodworth, M. D.

Annual Announcement of Long Island College Hospital, 1877–78.

---

The following is a complete list of exchanges received.

The Medical Record.
The London Lancet.
The Boston Medical and Surgical Journal.

Louisville Medical News.
The Clinic.
The Medical and Surgical Reporter.
Philadelphia Medical Times.
The American Medical Bi-Weekly.
The Hospital Gazette and Archives of Clinical Surgery.
Saint Louis Clinical Record.
Saint Louis Medical and Surgical Journal.
The American Practitioner.
Virginia Medical Monthly.
The Ohio Medical and Surgical Journal.
The Western Lancet.
Toledo Medical and Surgical Journal.
Nashville Journal of Medicine and Surgery.
The Sanitarian.
The Southern Medical Record.
The Cincinnati Lancet and Observer
The Obstetrical Journal.
Maryland Medical Journal.
The Chicago Medical Journal and Examiner.
The Cincinnati Medical News.
New York Medical Journal.
The Canada Medical Record.
The New Orleans Medical and Surgical Journal.
The Monthly Abstract of Medical Science.
The Medical News and Library.
Proceedings of the Medical Society of the County of Kings.
The Richmond and Louisville Medical Journal.
The Ohio Medical Recorder.
Atlanta Medical and Surgical Journal.
L'Union Medicale du Canada.
Detroit Medical Journal.
Pacific Medical and Surgical Journal
Monthly Journal of Southern Illinois Medicial Association.
The London Medical Press and Circular.
American Journal of the Medical Science.
Quarterly Journal of Inebriety.
Half-yearly Compendium of Medical Science.
Archives of Dermatology—July No.
A Series of American Clinical Lectures.
The Journal of Nervous and Mental Diseases.
Braithwaite's Retrospect.
American Journal of Insanity.
Hall's Journal of Health.
Boston Journal of Chemistry.
The Pharmacist.
American Journal of Pharmacy.
New Preparations.
Journal of Materia Medica.
American Journal of Dental Science.
The Dental Cosmos.
The Dental Advertiser.
The Missouri Dental Journal.
Chicago Medical Times.
The Medical Eclectic.
The Eclectic Medical Journal.
The Medical Review.
The American Medical Journal.
The Physio-Medical Journal.
The Cincinnati Medical Advance.
The United States Medical Investigator.
The New England Medical Gazette.
American Observer.
The Hahnemannian Monthly.
The Popular Science Monthly and Supplement.
Pen and Plow.
American Agriculturist.

### Contents of December Number.

### ORIGINAL.

ART. I.—Materia Medica and New Remedies. By Thomas D. Washburn, M. D., .......... 161
ART. II.—External Glandular Enlargements. By Z. Collins McElroy, M. D., .......... 168
ART. III.—Summary of Remarks on Fractures of the Shaft of the Femur in Adults. By Prof. Frank H. Hamilton. Reported by J. C. Lay, M. D., M. R. C. S. E., .......... 173

### CORRESPONDENCE.

THE BUFFALO

### Medical and Surgical Journal

PUBLISHED MONTHLY,

Containing Original Articles, Reports of Medical Societies and Hospitals, Editorial, Reviews, Correspondence, Army News, etc., etc; including the usual variety of Medical Periodical Publications. Specimen copies sent on application.

Address Buffalo Medical & Surgical Journal, Buffalo, N. Y.

TERMS OF SUBSCRIPTION, $3.00 PER YEAR, IN ADVANCE.

All communications for publication shoud be sent before the twentieth (20th) of each month, or they will of necessity lie over until the next number. No change can be made in advertisements after the twenty-fifth. Address, Buffalo Medical and Surgical Journal, 978 Main Street.

Correspondence and original articles are solicited. Publication is no evidence that the views of the author are endorsed.

